# The high concentration of progesterone is harmful for endometrial receptivity and decidualization

**DOI:** 10.1038/s41598-017-18643-w

**Published:** 2018-01-15

**Authors:** Yu-Xiang Liang, Li Liu, Zhi-Yong Jin, Xiao-Huan Liang, Yong-Sheng Fu, Xiao-Wei Gu, Zeng-Ming Yang

**Affiliations:** College of Veterinary Medicine, South China Agricultural University, Guangzhou, 510642 China

## Abstract

Progesterone is required for the establishment and maintenance of mammalian pregnancy and widely used for conservative treatment of luteal phase deficiency in clinics. However, there are limited solid evidences available for the optimal timing and dose of progesterone therapy, especially for the possible adverse effects on implantation and decidualization when progesterone is administrated empirically. In our study, mouse models were used to examine effects of excess progesterone on embryo implantation and decidualization. Our data indicate that excess progesterone is not only harmful for mouse implantation, but also impairs mouse decidualization. In excess progesterone-treated mice, the impaired LIF/STAT3 pathway and dysregulated endoplasmic reticulum stress may lead to the inhibition of embryo implantation and decidualization. It is possible that the decrease in birth weight of excess progesterone-treated mice is due to a compromised embryo implantation and decidualization. Furthermore, excess progesterone compromises *in vitro* decidualization of human endometrial stromal cells.

## Introduction

Embryo implantation and decidualization are tightly orchestrated by progesterone (P) and estrogen. P is essential for the maintenance of pregnancy in mammals^1^. P exerts its effects predominantly through the P receptor (PR), including two isoforms (PRA and PRB). PR deficiency in mice leads to pleiotropic reproductive abnormalities^2,3^. PRA deficiency results in severe uterine dysfunction and failure in ovulation, embryo implantation, and decidualization, whereas PRB knockout mice show normal ovarian and uterine function and fertile^4–6^. Ovariectomy results in abortion because ovary is the main source of P during early pregnancy until formation of a functional placenta. Pregnancy can be rescued by P supplementation in ovariectomized pregnant rats^7^. P supplementation can also extend uterine receptivity through day 6 of pseudo-pregnancy in mice^8^. Similarly, P injection maintains the plasma P level and rescues the pregnancy in lutectomized women^9^.

The potential for implantation is compromised if there is a decrease in the amount or duration of P production by the corpus luteum, or if there is a poor endometrial response to P^10^. In the human, administration of P receptor antagonists within the first 7 weeks of pregnancy results in abortion^11^. A low dose of mifepristone is able to inhibit human embryo implantation process in a three-dimensional co-culture system^12^.

On the one hand, luteal phase deficiency (LPD), defined as a condition of insufficient P secretion to maintain a normal secretory endometrium and support for successful embryo implantation and development^13^. Several lines of factors are contributing to the etiology of LPD, including the removal of large quantities of granulosa cells during the oocyte retrieval, human chorionic gonadotropin (hCG) administration for superovulation, and other superovulation regimens in stimulated IVF cycles, although the debate on this topic remains unsolved^14^. The diagnosis criterion of LPD is still confusing up to date^15^. For conservative treatment of suspected LPD patients, P is routinely recommended for luteal phase support. However, a prospective study fails to demonstrate any positive effect of P on the pregnancy outcome of threatened abortion^16^.

Even if supplementation with P may reduce the incidence of recurrent miscarriages, it is difficult to recommend the route and dose of P therapy^17^. Besides, there is no evidence indicating that P is beneficial to natural and unstimulated cycles^13^. More studies should be done to determine the optimal dosage and possible adverse effects on implantation and decidualization when P is used empirically in clinical trial for luteal phase support^18^.

On the other hand, ovarian stimulation program is routinely used to induce multiple ovulation in human *in vitro* fertilization (IVF), which inevitably leads to ultra-physiological level of P on the day of hCG administration, defined as ‘premature luteinization’. Premature luteinization may be caused by multiple follicles, the overdose of gonadotropins and poor ovarian response. The frequency of elevated serum P level varies between 5% and 38% due to the discrepancy on stimulation regimen, method of P assessment and P cut-off level^19^. It is still controversial whether the high P serum level at the end of follicular phase has any adverse impacts on ongoing pregnancy outcome^20^. Several studies show that there is no significant difference on IVF pregnancy outcome between normal and high P serum level (≥0.9 ng/ml) on hCG day^21,22^. However, premature P elevation (≥1.5 ng/ml) in stimulated IVF cycles seems to have a detrimental influence on the pregnancy outcome^23–25^. Therefore, further evidences are badly needed to clarify these controversial issues.

In this study, the effects of P at different concentrations on embryo implantation and decidualization were evaluated in mouse models. Effects of excess P on human *in vitro* decidualization were also examined. Our data suggested that endometrial receptivity and decidualization are compromised by a high level of P in mice, and human *in vitro* decidualization is also impaired by supplementation of excess P.

## Results

### Effects of excess P on mouse endometrium receptivity

**Figure 1:**
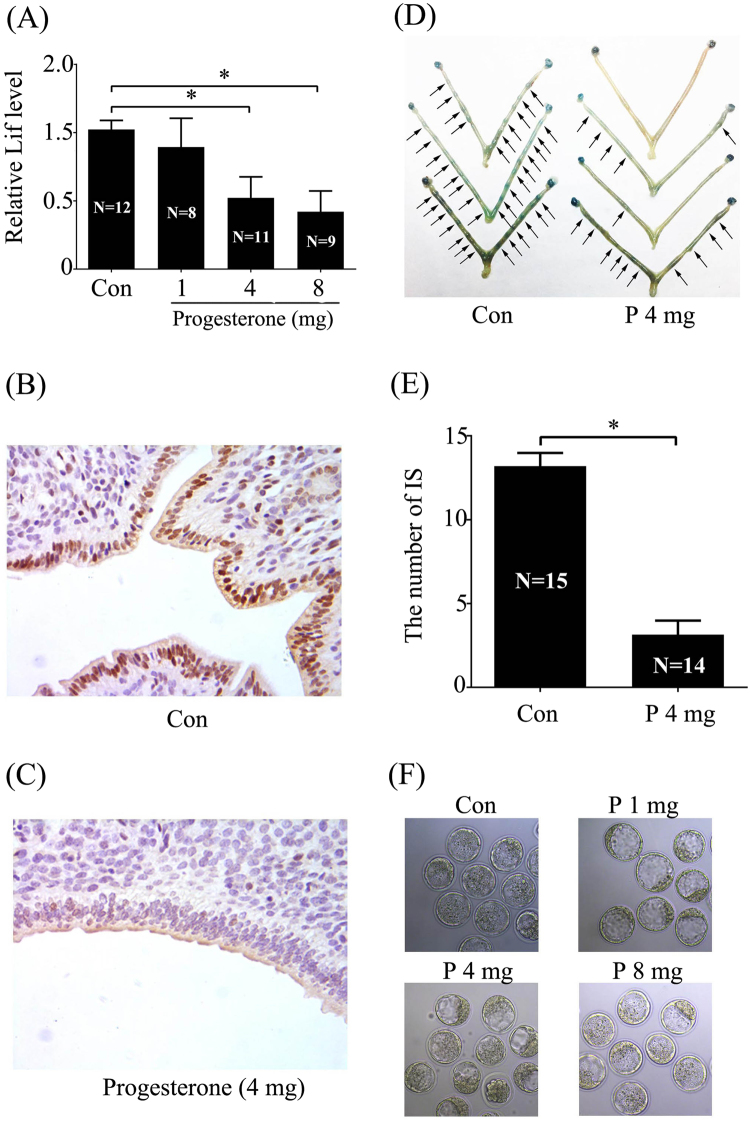
Effects of excess P on embryo implantation and implantation-related genes. (**A**) The mRNA expression of Lif in mouse day 4 uteri treated with oil or different concentrations of P on D3 9:00. A representative photograph showing the protein expression of p-Stat3 in mouse day 4 uteri treated with (**B**) oil or (**C**) 4 mg P on D3 9:00. (**D**) A representative photograph showing the number of implantation sites (IS) at day 4 midnight following treatments with oil or 4 mg P twice (D3 9:00 and D4 9:00). (**E**) The quantification of implantation sites in day 4 midnight. (**F**) The morphology of blastocysts from those mice treated with oil or different concentrations of P. The real-time values are normalized to the Rpl7 expression level and indicated as the mean ± SEM. n = 3. *P < 0.05.

Leukemia inhibitory factor (LIF) is strongly expressed in the glandular epithelium and required for mouse implantation^26^. The phosphorylation of Stat3, as a receptivity marker on day 4 of pregnancy in mice, is at the downstream of LIF^27^. Therefore, pregnant mice were treated with 1,4 and 8 mg P/mouse on day 3 9:00, compared to control, the level of LIF mRNA expression on day 4 9:00 was inhibited by 4 or 8 mg/mouse P (Fig. 1A). Accordingly, the level of phosphorylated Stat3 in the luminal epithelium was sharply decreased on days 4 of pregnancy after day 3 pregnant mice were treated with 4 mg P /mouse, (Fig. 1B and C). When pregnant mice are treated with 4 mg P/mouse on days 3 and 4, the number of implantation sites are significantly reduced compared to control at midnight on day 4 of pregnancy (Fig. 1D and E). To evaluate whether excess P has any negative effect on embryo development, then we examined the blastocyst development at the 14:00 of day 4. The morphology of blastocysts from different dose of P treated mice is normal and similar to vehicle control (Fig. 1F).

### Effects of excess P on the expression of PR, estrogen receptor (ER) and P target genes

**Figure 2:**
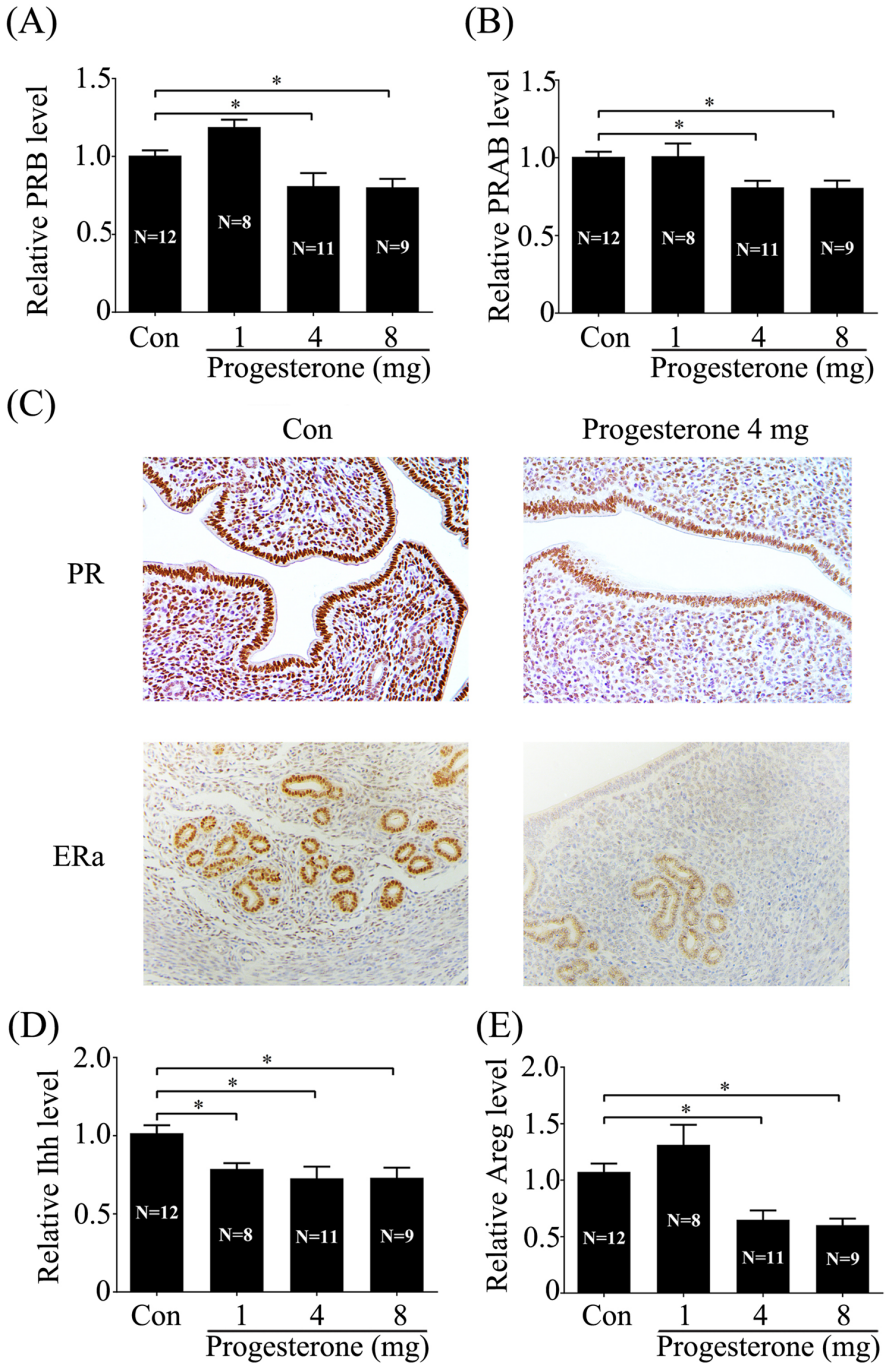
Effects of excess P on the expression of PR, ER and P target genes after day 3 pregnant mice were treated with oil or different concentrations of P for 24 h. (**A**) The mRNA expression of PRB. (**B**) The mRNA expression of PRAB. (**C**) A representative photograph showing PR and ER immunostaining. (**D**) The mRNA expression of Ihh in mouse uteri. (**E**) The mRNA expression of Areg in mouse uteri. The real-time values are normalized to the Rpl7 expression level and indicated as the mean ± SEM. n = 3. *P < 0.05.

Because P executes its function through PR, effects of excess P on PR expression were examined. The levels of total PRB and PRAB expression were reduced by 4 or 8 mg/mouse P, not by 1 mg/mouse P (Fig. 2A and B). Compared to control, the level of PR immunostaining was also decreased by 4 mg P/mouse (Fig. 2C). In mouse uterus, P inhibits estrogen-induced cell proliferation^28^. Therefore, effects of excess P on ER were also examined. Compared to control, ER immunostaining was slightly inhibited in 4 mg P-treated mouse uterus (Fig. 2C).

Ihh and Areg are P target genes and essential for mouse embryo implantation^29–31^. When day 3 pregnant mice were treated with different concentrations of P, the levels of both Ihh and Areg were obviously downregulated by 4 or 8 mg/mouse P. Ihh expression was also reduced by 1 mg/mouse P (Fig. 2D and E).

### Effects of excess P on mouse decidualization and birth weight

**Figure 3:**
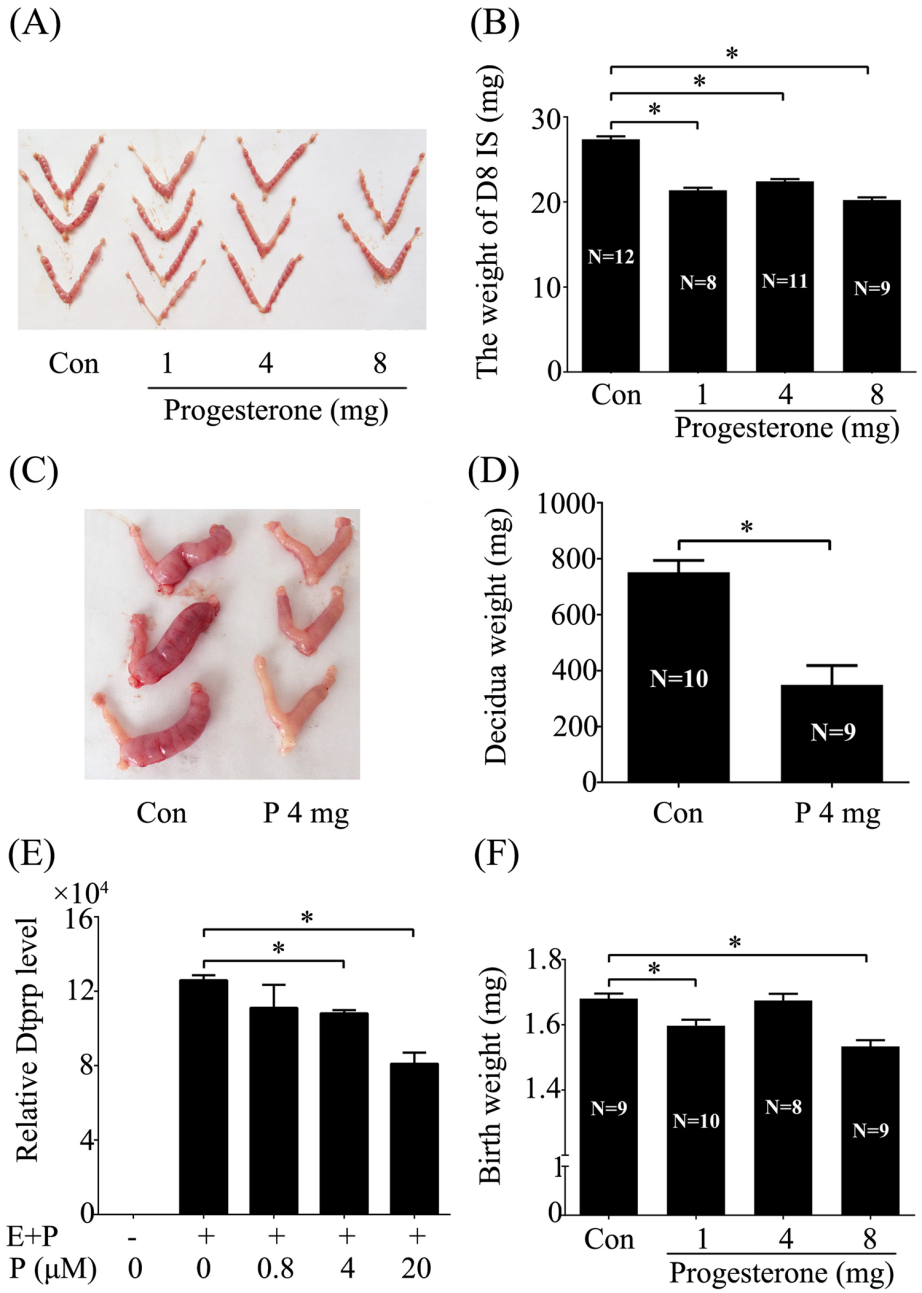
Effects of excess P on mouse decidualization and birth weight. (**A**) A representative photograph showing mouse day 8 uteri treated with oil or P (1,4 and 8 mg/mouse) daily from days 3 to 7. (**B**) The average weight of implantation sites on day 8 mice treated with oil or P (1,4 and 8 mg/mouse) daily from days 3 to 7. (**C**) A representative photo showing the deciduoma on day 8 pseudo-pregnant mice under artificial decidualization treated with oil or P (1,4 and 8 mg/mouse) daily from days 5 to 7. (**D**) The deciduoma weight on day 8 after pseudo-pregnant mice under artificial decidualization were treated with oil or P (1,4 and 8 mg/mouse) daily from days 5 to 7. (**E**) Effects of excess P on the expression of Dtprp, a marker for mouse *in vitro* decidualization. (**F**) Effects of excess P on the birth weight after pregnant mice were treated with oil or P (1,4 and 8 mg/mouse) daily from days 3 to 7, the weights of individual newborn fetus were counted. The real-time values are normalized to the RPL7 expression level and indicated as the mean ± SEM. n = 3. *P < 0.05.

In order to analyze effects of excess P on mouse decidualization, day 3 pregnant mice were treated with different doses of P daily (from days 3 to7). Compared to control, the weight of implantation sites on day 8 was significantly declined by 1, 4 and 8 mg/mouse P (Fig. 3A and B). In order to exclude effects of excess P on embryonic development, pseudo-pregnant mice under artificial decidualization were treated with 4 mg/mouse P. Treatment of 4 mg/mouse P caused a significant decrease on the weight of deciduoma (Fig. 3C and D). To further verify effects of excess P on mouse decidualization, mouse stromal cells under *in vitro* decidualization were treated with P. Under *in vitro* decidualization, Dtprp, a marker for mouse decidualization^32^, was significantly induced, while Dtprp expression was significantly suppressed by 4 and 20 μM, but not by 0.8 μM P (Fig. 3E).

Because treatment of excess P during early pregnancy had significant effects on embryo implantation and decidualization, we would like to explore whether these effects during early pregnancy affect the whole pregnant outcome. After day 3 pregnant mice were treated with 1, 4 and 8 mg/mouse P daily for 5 days from days 3 to 7, respectively, the birth weight of P treated mice was significantly reduced by 1 and 8 mg/mouse P, not by 4 mg/mouse P (Fig. 3F).

### Effects of excess P on the expression of P and estrogen target genes in ovariectomized mice

**Figure 4:**
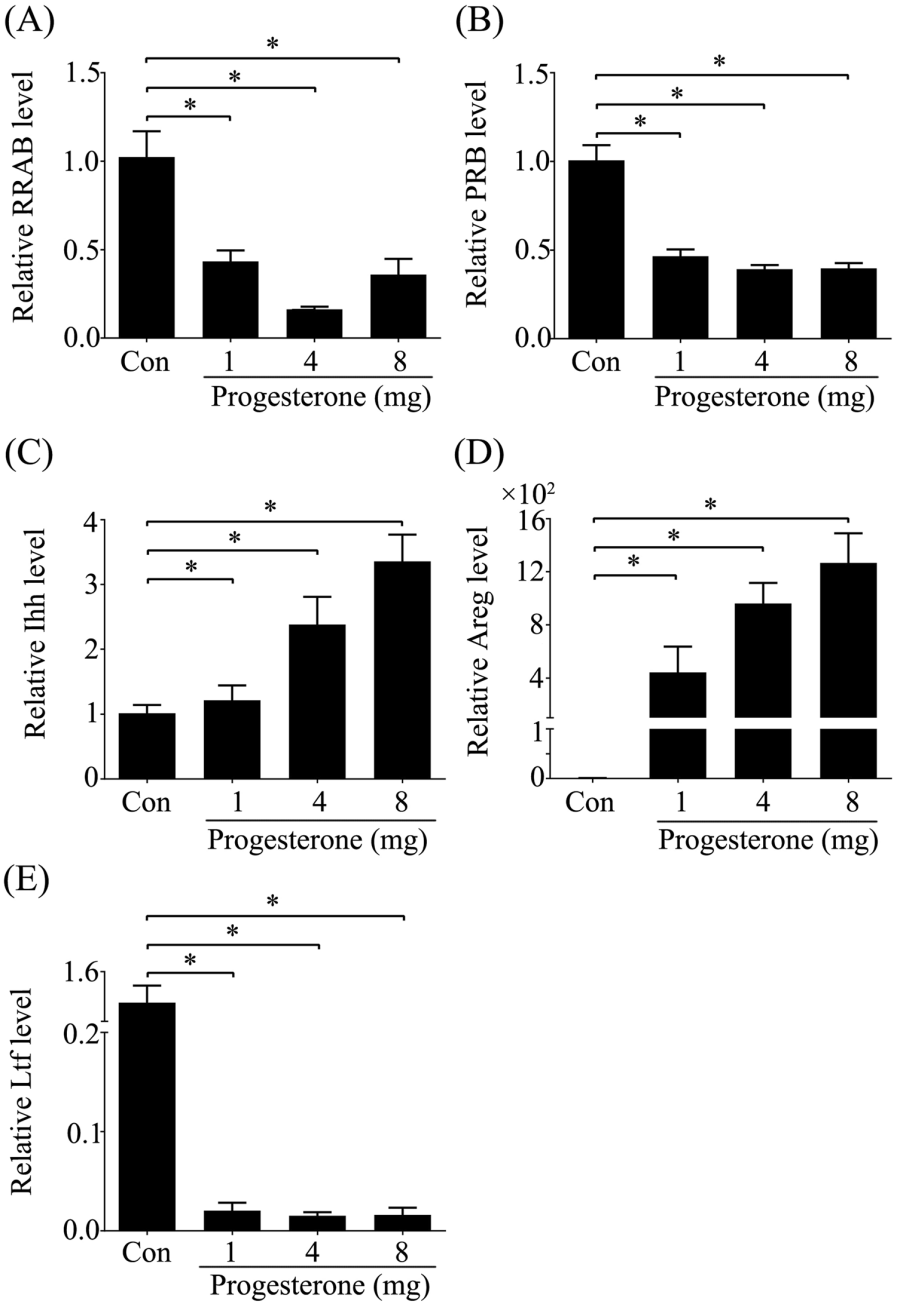
Effects of excess P in ovariectomized mice. Ovariectomized mice were treated with oil or different concentrations of P daily for 3 days and the relative mRNA expression of these genes to GAPDH were detected by real-time PCR. (**A**) PRAB. (**B**) PRB. (**C**) Ihh. (**D**) Areg. (**E**) Ltf. The real-time values are normalized to the Rpl7 expression level and indicated as the mean ± SEM. n = 3. *P < 0.05.

After ovariectomized mice were treated with different concentrations of P daily for 3 days, real time PCR was performed to analyze gene expression. The expression levels of total PRAB (Fig. 4A) and PRB (Fig. 4B) were significantly inhibited by different concentrations of P. Both of Ihh (Fig. 4C) and Areg (Fig. 4D) were up-regulated by 2, 4 and 8 mg/mouse P. However, estrogen target gene LTF was significantly suppressed by different concentrations of P (Fig. 4E).

### Effects of excess P on endoplasmic reticulum stress

**Figure 5:**
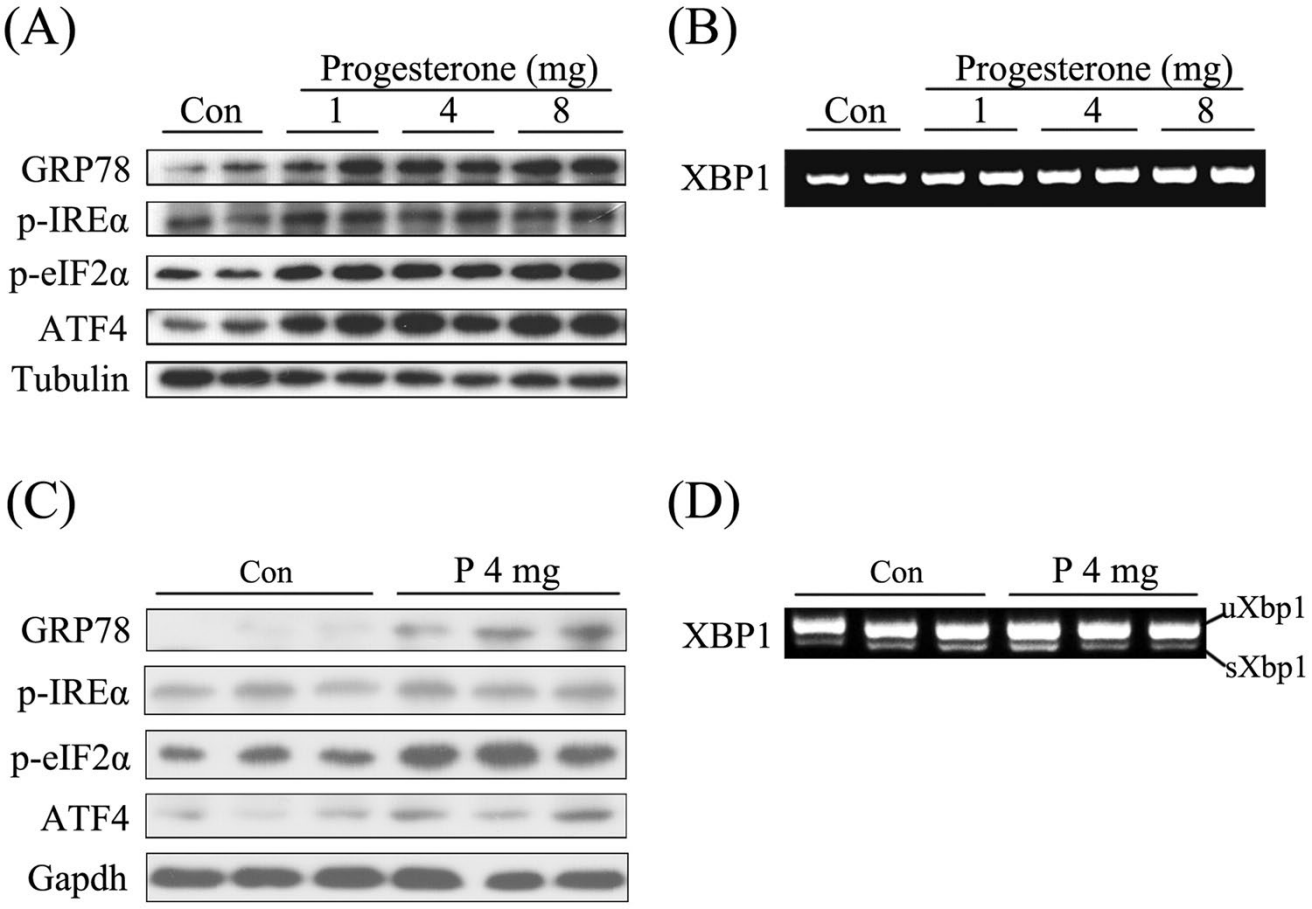
Effects of excess P on endoplasmic reticulum stress. (**A**) The protein expressions of GRP78, p-IRE1α, p-eIF2a and ATF4 of ovariectomized mice treated with oil or different concentrations of P daily for 3 days were detected by Western blot. (**B**) The mRNA expression of XBP1 of implantation sites in mice treated with oil or 4 mg/mouse P daily for 3 days were detected by agarose gel electrophoresis. (**C**) GRP78, p-IRE1α, p-eIF2a and ATF4 levels of implantation sites in mice treated with oil or 4 mg/mouse P daily from days 3 to 7 were detected by Western blot. (**D**) The mRNA expression of spliced and un-spliced XBP1 of implantation sites in mice treated with oil or 4 mg/mouse P daily from days 3 to 7 were detected by agarose gel electrophoresis.

ER stress is shown to be required for mouse decidualization^33^. Ovariectomized mice were treated with different concentrations of P to examine its effects on ER stress. In P-treated uterus, endoplasmic reticulum stress was activated, especially for GRP78/p-eIF2a/ATF4 pathway (Fig. 5A). P treatment had little effects on IRE1a/XBP1 pathway. Spliced XBP1 (sXBP1) mRNA level didn’t show obvious changes following P treatments (Fig. 5B). Previous investigation indicated that GRP78/IREα/XBP1 pathway is physiologically activated in mouse decidualization^33^. Then in order to analyze if excess P has any effect on ER stress of day 8 uteri, day 3 pregnant mice were treated with 4 mg/mouse P daily from days 3 to 7 for 5 days, we found that GRP78/eIF2a/ATF4 pathway was aberrantly upregulated by excess P in decidua of day 8 (Fig. 5C). Similarly, excess P didn’t show any obvious effects on IRE1a/XBP1 pathway because spliced Xbp1 remained unchanged following P treatments (Fig. 5D).

### Effects of excess P on human *in vitro* decidualization

**Figure 6:**
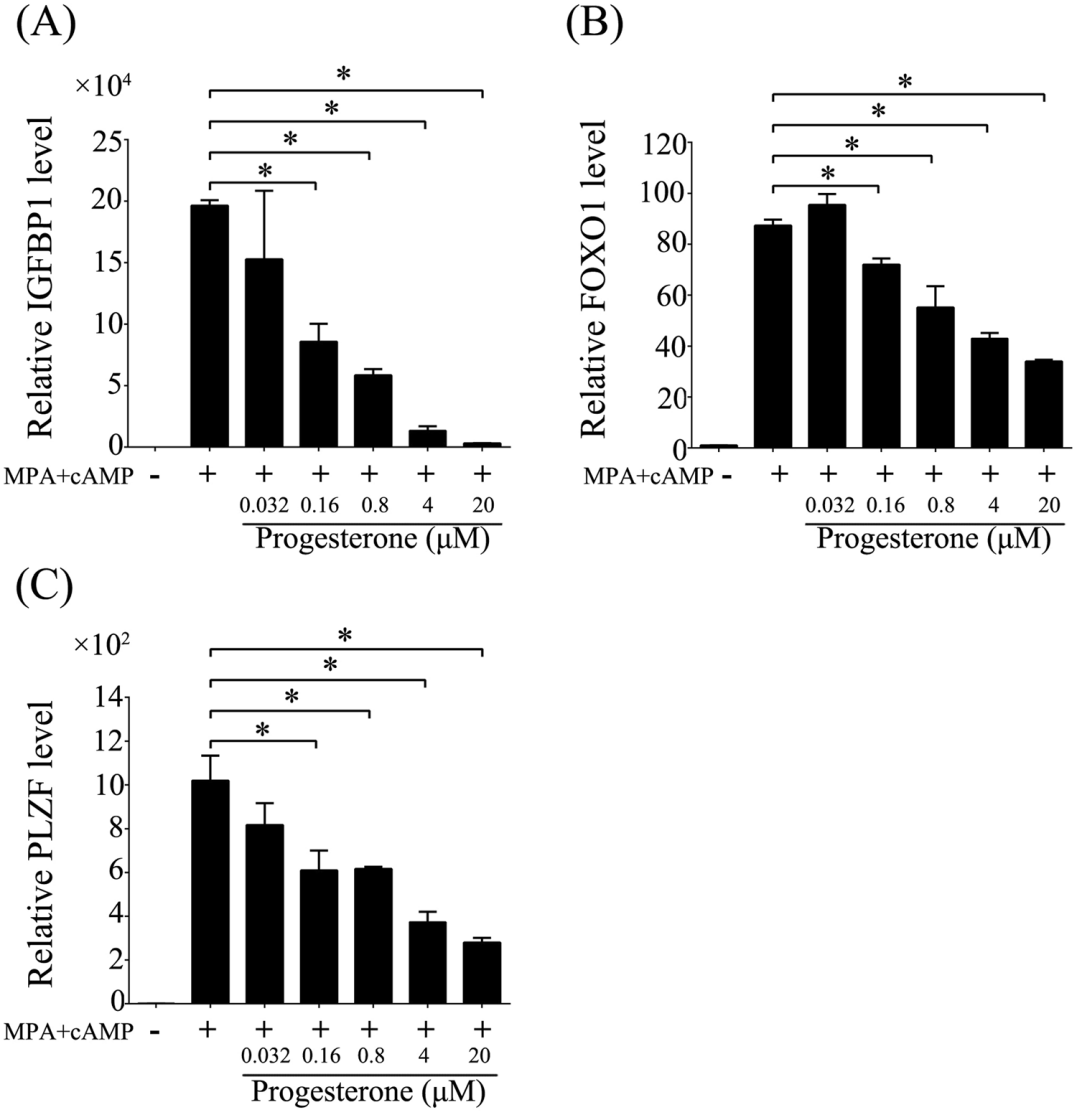
Effects of excess P on human *in vitro* decidualization. The relative mRNA expressions of decidualization marker genes were examined by real time PCR. (**A**) IGFBP1. (**B**) FOXO1. (**C**) PLZF. The values are normalized to the GAPDH expression level and indicated as the mean ± SEM. n = 3. *P < 0.05.

In mice, we showed that decidualization was impaired by excess P treatments. Then we would like to examine effects of excess P on human *in vitro* decidualization. Under human *in vitro* decidualization, there was a significant increase for the expression levels of IGFBP-1^34^, FOXO1^35^ and PLZF^36^, the well-known markers for human *in vitro* decidualization. At the same time, the expression levels of IGFBP-1, FOXO1 and PLZF were significantly suppressed by excess P in a dosage-dependent manner (Fig. 6A–C).

## Discussion

The endometrium is a highly hormone responsive tissue. Under the influence of steroid sex hormones, the endometrium undergoes dynamic changes prepared for embryo implantation and decidualization^37^. Estrogen and P are the major mediators for embryo implantation and decidualization^38^. Estrogen is critical for determining the duration of implantation window. A high level of estrogen will lead to the close of implantation window^39^. Estrogen administration during early gestation can disrupt implantation^40^. The increased estrogenic responses caused from uterine deletion of gp130 or Stat3 also result in implantation failure^41^. Although effects of excess estrogen on receptivity and decidualization have been extensively explored, whether excess P affects receptivity and decidualization remains to be clarified. P is widely used to treat women with threatened abortion for maintaining pregnancy. However, up to now, there is no real consensus on the timing, dose and routes of P administration^42,43^.

LPD ubiquitously exists in IVF with controlled ovarian hyper-stimulation and other pathological conditions. The aetiology and diagnosis criterion of LPD are still not well established in current clinics^15,44^. It is empirical for clinicians to treat all the possible LPD with P for that they may ignore the adverse effects of excessive supplementation of P on pregnancy. Almost all of current evidence suggests that luteal phase support using P can play positive effects on main pregnancy outcome^43^. While studies conducted by Kyrou *et al*. don’t show any improvement of pregnancy rates in women received routine P supplementation^45,46^. In spite of the potential risks for excess P supplementation, little attention has been paid to the possible detrimental impacts of excess P supplementation on pregnancy outcome. Our data showed that excessive P dramatically destroys decidualization process in mice and humans in a dosage-dependent manner, which may give a reference on the clinical use dosage of P. The side-effects of excessive administration of P on the reproduction outcomes should be carefully taken into consideration.

There are accumulating evidences indicating that premature P over 1.5 ng/ml in stimulated IVF cycles seems to have detrimental influences on the pregnancy outcome^23–25^. High P level (1.7 ng/ml) before oocyte retrieval is associated with an obvious reduction of endometrial receptivity^47^. The gene expression profile of the endometrium is indeed affected when P level is above 1.5 ng/ml at the end of the follicular phase^48^. Elevated P levels on the day of hCG during the initial fresh cycle are correlated with poor pregnancy in the fresh transfer cycles but not in subsequent frozen-thawed embryo transfer cycles^49^. A previous retrospective study including 4,106 IVF/intracytoplasmic sperm injection cycles reported that patients with P ≥ 2 ng/mL exhibit more high-quality embryos than patients with P < 1 ng/mL^50^, while our data show that excess P has no detrimental effect on embryo development, which suggests that it is endometrium, not the oocyte, that is compromised by P elevation. In a recent study, women in GnRH down-regulation cycles were treated with different dose of P (2.5, 5, 10, 40 mg/day) and compared to control group female with normal ovulation. The high dose of P (≥5 mg/day) is harmful for endometrium receptivity due to aberrant gene expression in spite of a normal histology^51^. Our data also showed a harmful impact on mouse receptivity and decidualization in excess P group, in addition to impaired decidualization of human endometrial stromal cells *in vitro*.

In our study, excess P-treated mice exhibit a lower birth weights than control, which is in line with previous studies that neonatal birth weights are lower in fresh blastocyst transfer cycles after controlled ovarian stimulation than in frozen-thawed embryos transfer cycles without ovarian stimulation^52,53^. Another latest retrospective analysis indicates that in fresh embryo transfers cycle, patients with elevated P levels (>2.0 ng/mL) suffer from lower birth weight compared to P levels ≤ 2.0 ng/mL counterparts^54^. The cumulating evidences indicate that it is not advisable to perform embryo transfer for patients with high levels of P in fresh cycle.

Successful implantation requires a synchronous cross-talk between a competent blastocyst and a receptive endometrium^55^. The primary masters that coordinate the endometrium receptivity are estrogen and P^56^. In natural mouse reproduction cycle, uterine epithelial cell proliferation is stimulated by a pre-ovulatory estrogen. P secreted from newly formed corpus luteum enhances uterine stromal cell proliferation. The combined actions of P4 and estrogen is required to establish receptivity for implantation^39,57^. In our study, Lif expression and p-Stat3 immunostaining were inhibited by excess P. LIF expression is also downregulated following ablation of epithelial PR or PRA overexpression in in whole uterus or uterine epithelium^58,59^. However, ER immunostaining was reduced by excess P. These evidences suggest that the downregulation of PR and ER may contribute to the decrease of Lif expression.

As an estrogen-responsive gene, LIF is crucial for uterine receptivity and implantation, for that its deletion leads to implantation failure in mice^26,60^. There is a decline of LIF in endometrial glandular epithelium of women with recurrent implantation failure after IVF^61^. As a direct downstream target of LIF, signal transducer and activator of transcription 3 (STAT3) is phosphorylated during the establishment of uterine receptivity^27^. Implantation is impaired when STAT3 phosphorylation is inhibited or uterine conditional deletion of STAT3 is performed^41,62^. It is reported that endometrial p-STAT3 is reduced in some women with unexplained infertility^63^. In our study, a significant reduced expression of LIF and p-STAT3, resulted from excess P treatment, may underline the deficiency in embryo implantation.

Indian hedgehog (Ihh) has been identified as a target gene of P and is expressed in epithelium, mediating epithelial-mesenchymal interactions in the mouse uterus. Conditional knockout of Ihh in the murine uterus results in infertility for defective embryo implantation and decidualization^64^. Another P regulated gene, amphiregulin (Areg), has also been identified as a receptivity marker for implantation^29^. In our study, Ihh and Areg are conspicuously down-regulated by excess P in mouse uterus on day 4 pregnancy, indicating a compromised endometrium receptivity.

Perturbation of endoplasmic reticulum (ER) protein homeostasis leads to the accumulation of misfolded proteins in its lumen and subsequently causes a stress that is called ER stress, consisting of three pathways: glucose regulated protein 78 (GRP78)/inositol requiring enzyme 1 a (IRE1a)/X-box protein 1(XBP1) signaling pathway, activating transcription factor 6(ATF6) pathway and pancreatic ER kinase (PERK)/eukaryotic translation initiation factor 2a (eIF2a)/activating transcription factor 4 (ATF4) pathway^65^. GRP78/IRE1a/XBP1 pathway is activated and essential during mouse decidualization. excessive or chronic ER stress is harmful to mouse decidualization^33^. Sustained endoplasmic reticulum stress-induced apoptosis in decidualization may play an ignominious role in early pregnancy loss^66^. The markers of ER stress, GRP78, IRE1a, and spliced XBP1 (sXBP1), are significantly increased in fetal membranes and myometrium after term and preterm labor^67^. Excessive potentiation of uterine ER stress fails to maintain uterine caspase-3 and 7 levels, leading to preterm birth^68^. Our results showed that excess P can promote ER stress by predominantly up-regulating GRP78/eIF2a/ATF4 pathway in ovariectomized mice. Similarly, GRP78/eIF2a/ATF4 pathway is activated by excess P4 in day 8 pregnant mouse uterus, resulting in a repression of decidualization. In humans, developmentally impaired embryos elicit an anomalous endoplasmic stress response in human decidual cells^69^. PERK/eIF2a and ATF6 signaling pathway is activated in fetal growth restriction^70^. Actually, in present study, mice received excess P deliver a lower birth weight, with an aberrant elevation of GRP78/eIF2a/ATF4 signaling at implantation sites on day 8 pregnancy.

In conclusion, our study indicates that excess P has a detrimental effect on endometrium receptivity and decidualization. Excess P treatment may cause fetal growth restriction through compromising embryo implantation and decidualization in mouse models.

## Materials and Methods

### Animal Treatments

All animal experiments were approved by Animal Care and Use Committee of South China Agricultural University. All of the experiments were carried out in accordance with the approved guidelines by South China Agricultural University. Adult CD1 mice were housed in a temperature- and light-controlled environment with 14 h light: 10 h dark cycle. Pregnant or pseudo-pregnant female mice (8-10 weeks) were obtained by mating with fertile or vasectomized males of the same strain (day 1 is the day of vaginal plug), respectively. From days 1–4, pregnancy was verified by flushing the embryos from the fallopian tube and uterus, respectively. The implantation sites on day 5 were confirmed by tail intravenous injection of Chicago blue dye (Sigma). Artificial decidualization was performed as previously described^71^.

In order to examine the effects of P on the expression of endometrial receptivity-related genes, pregnant mice were subcutaneously injected with 1, 4, and 8 mg /mouse P (Sigma, dissolved in 100 μl sesame oil) at 9:00 on day 3 of pregnancy. The control mice received 100 μl of sesame oil. Mice were sacrificed at 9:00 on day 4 of pregnancy to collect uteri for further analysis.

For examining the effects of excess P on implantation sites, pregnant mice were subcutaneously injected with 4 mg/mouse P (Sigma, dissolved in 100 μl sesame oil) twice (9:00 on day 3 and 9:00 on day 4). The control mice received 100 μl sesame oil/mouse. Mice were sacrificed at midnight on day 4 to collect uteri for counting implantation sites. Blastocysts were flushed from uterine horns of mice at 14:00 on day 4 of those mice.

To analyze the effects of excess P on the weight of mouse implantation sites on day 8, pregnant mice were subcutaneously injected with 1, 4, and 8 mg P/mouse (Sigma, dissolved in 100 μl sesame oil) daily from 9:00 on day 3 to 9:00 on day 7 for 5 days. The control mice received 100 μl sesame oil/mouse. Mice were sacrificed at 9:00 on day 8 to collect uteri for weighing implantation sites.

To exclude the effects of P on embryonic development, pseudo-pregnant mice induced for artificial decidualization were treated with a daily injection of 4 mg /mouse P (dissolved in 100 μl sesame oil) on days 5, 6 and 7 of pseudo-pregnancy. Uteri were collected and weighed on day 8 of pseudo-pregnancy.

Ovariectomized mice were subcutaneously injected with 1, 4, and 8 mg /mouse P (Sigma, dissolved in 100 μl sesame oil) daily for 3 days. The control mice received 100 μl sesame oil. Mice were sacrificed 24 h after last injections to collect uteri for further analysis.

### Immunohistochemistry

Immunohistochemistry was performed as described previously^72^. Briefly, paraffin-embedded uterine sections were deparaffinized in xylene, rehydrated through a graded series of ethanol, and washed in water. Antigen retrieval was performed in 0.01 M sodium citrate buffer (pH 6.0) by microwaving for 10 min. Endogenous horseradish peroxidase (HRP) activity was inhibited with 3% H_2_O_2_ for 15 min. After blocked with 10% horse serum at 37 °C for 1 h, sections were incubated with rabbit anti-PR (1:1200, #MA5-14505, Thermo Fisher Scientific, MA, USA), rabbit anti-ER (1:2000, #sc-7207, Santa Cruz Biotechnology, TX USA), rabbit anti-p-Stat3 (1:400, #9145, Cell Signaling Technology, MA, USA), diluted in 10% horse serum at 4 °C overnight, respectively. Followed by washing and incubating with biotin-labeled goat anti-rabbit IgG antibodies (Zhongshan Golden Bridge, Beijing, China) for 30 min, then sections were incubated with streptavidin-HRP complex (Zhongshan Golden Bridge, Beijing, China) for 30 min. The positive signals were visualized using DAB Horseradish Peroxidase Color Development Kit according to the manufacturer’s protocol (Zhongshan Golden Bridge, Beijing, China). The sections were counterstained with hematoxylin.

### Isolation and treatment of mouse endometrial stromal cells

Primary endometrial stromal cells were enzymatically isolated from day 4 pregnant mice and cultured as described previously^71^. Briefly, mouse uteri were digested with Hanks’ balanced salt solution (Sigma) containing 1% trypsin (AMRESCO) and 6 mg/ml dispase (Roche). Luminal epithelial cells were removed after HBSS washing. After the remaining uteri were treated with 0.15 mg/ml collagenase I (Invitrogen), endometrial stromal cells were collected and cultured in DMEM/F12 (Sigma) containing 10% charcoal-treated FBS (Biological Industries, Israel). For inducing *in vitro* decidualization, primary endometrial stromal cells were treated with 10 nM estradiol-17β and 1 μM P. Under *in vitro* decidualization, cultured stromal cells were treated with 0.8, 4 and 20 μM P (Sigma), respectively. The highest treatment dose of P has no toxic effect on cell viability.

### Culture and *in vitro* decidualization of human endometrial stromal cells

Immortalized human endometrial stromal cells (hESC) were purchased from the American Type Culture Collection (ATCC CRL-4003TM) and cultured according to the manufacturer’s instructions^73^. Briefly, stromal cells were cultured in DMEM/F12 (Sigma) supplemented with 10% charcoal-stripped FBS (CS-FBS, Biological Industries) at 37 °C in a humidified chamber with 5% CO2. To induce decidualization *in vitro*, stromal cells were treated with 1 μM Medroxyprogesterone 17-acetate (MPA, Sigma) and 0.5 mM dibutyryl cAMP (db-cAMP, Sigma) in DMEM/F12 with 2% CS-FBS for 6 days. The medium was changed every 48 h. Under *in vitro* decidualization, stromal cells were treated with 0.032, 0.16, 0.8, 4, and 20 μM P (Sigma) for further analysis, respectively. The highest treatment dose of P has no significant toxic effect on cell viability.

### Real-time PCR and detection of spliced XBP1

Real-time PCR was performed as previously described^74^. Briefly, total RNAs from each sample were isolated using TRIzol reagent kit (Invitrogen), digested with RQ1 deoxyribonuclease I (Promega, Fitchburg, WI) and reverse-transcribed into cDNA with PrimeScript reverse transcriptase reagent kit (TaKaRa). For real time PCR, cDNA was amplified using a SYBR Premix Ex Taq kit (TaKaRa) on the CFX96 Touch™ Real-Time System (Bio-Rad). Data from real-time PCR were analyzed using the 2^△△Ct^ method and normalized to Rpl7 or GAPDH expression.

XBP1 primers were designed to contain the 26 base pairs which were used to detect the spliced and un-spliced XBP1 mRNA. PCR products were separated on 2.5% agarose gel electrophoresis as described previously^75^. The corresponding primer sequences were provided in Table 1.

**Table 1:** Primers used in this study.

Gene	Primer sequences (5′-3′)	Accession number	Size (bp)	Application
Ihh	GCTGAAGGGACTCTAACC ACAGAGGACGGAGACAAC	NM_010544.2	118	Real-time PCR
LTF	AGCCAACAAATGTGCCTCTTC CCTCAAATACCGTGCTTCCTC	NM_008522	119	Real-time PCR
LIF	AAAAGCTATGTGCGCCTAACA GTATGCGACCATCCGATACAG	NM_008501	98	Real-time PCR
Areg	CTCCACAGGGGACTACGACTA CTTGGGCTTAATCACCTGTTC	NM_009704.3	105	Real-time PCR
Hand2	GCTACATCGCCTACCTCA CCTTCTTCCTCTTCTCCTCT	NM_010402.4	114	Real-time PCR
PRB	GTCTTTGTAGTATTTACGGGTGCG AGCCCATTCTTACTCGTTCTCCT	NM_008829.2	153	Real-time PCR
PRAB	TATACCGATCTCCCTGGACG CCCTATGAGTGGCTTCTACC	NM_008829.2	137	Real-time PCR
RPL7	GCAGATGTACCGCACTGAGATTC ACCTTTGGGCTTACTCCATTGATA	M29016	129	Real-time PCR
Dtprp	AGCCAGAAATCACTGCCACT TGATCCATGCACCCATAAAA	NM_010088	119	Real-time PCR
Xbp1	GAGCAGCAAGTGGTGGATTT AAAGGGAGGCTGGTAAGGAA	NM_013842.3	447/421	Real-time PCR
IGFBP1	CCAAACTGCAACAAGAATG GTAGACGCACCAGCAGAG	NM_000596.2	87	Real-time PCR
FOXO1	CGAGCTGCCAAGAAGAAA TTCGAGGGCGAAATGTAC	NM_002015	105	Real-time PCR
PLZF	TCACATACAGGCGACCACC CTTGAGGCTGAACTTCTTGC	NM_006006.4	144	Real-time PCR
GAPDH	GAAGGTGAAGGTCGGAGT GATGGCAACAATATCCACTT	BC023632	94	Real-time PCR
RPL7	CTGCTGTGCCAGAAACCCTT TCTTGCCATCCTCGCCAT	NM_000971	194	Real-time PCR

### Western blot analysis

Western blot was performed as previously described^76^. Briefly, protein lysates were separated by SDS polyacrylamide gel electrophoresis and transferred onto a PVDF membrane. Membranes were incubated overnight at 4 °C with each primary antibody, including anti-GRP78 (sc-1050, Santa Cruz Biotechnology, TX USA), anti-IREa (#3294, Cell Signaling Technology, MA USA), anti-p-IRE1α (#ab48187, Abcam, UK), anti-p-eIF2α (#3398, Cell Signaling Technology, USA), anti-ATF4 (sc-200, Santa Cruz Biotechnology, TX USA), anti-p-Stat3 (#9145, Cell Signaling Technology, MA USA), anti-GAPDH (sc-25778, Santa Cruz Biotechnology, TX USA) and anti-Tubulin (#2144, Cell Signaling Technology, MA USA). Then the membrane was incubated in 5% non-fat milk containing HRP-conjugated secondary antibody (1:5000) for 1 h. Signals were detected by ECL Chemiluminescent kit (Millipore).
